# Large magnetocaloric effect in manganese perovskite La_0.67−*x*_Bi_*x*_Ba_0.33_MnO_3_ near room temperature

**DOI:** 10.1039/c8ra09802f

**Published:** 2019-02-13

**Authors:** Ah. Dhahri, E. Dhahri, E. K. Hlil

**Affiliations:** Laboratoire de Physique Appliquée, Faculté des Sciences de Sfax, Université de Sfax BP 1171 3000 Tunisia dhahridhahri14@gmail.com +216 20 20 45 55; Faculté des Sciences de Monastir Avenue de l'environnement 5019 Monastir Tunisia; Institut Néel, CNRS et Université J. Fourier BP 166 38042 Grenoble France

## Abstract

La_0.67−*x*_Bi_*x*_Ba_0.33_MnO_3_ (*x* = 0 and 0.05) ceramics were prepared *via* the sol–gel method. Structural, magnetic and magnetocaloric effects have been systematically studied. X-ray diffraction shows that all the compounds crystallize in the rhombohedral structure with the *R*3̄*c* space group. By analyzing the field and temperature dependence of magnetization, it is observed that both samples undergo a second order magnetic phase transition near *T*_C_. The value of *T*_C_ decreases from 340 K to 306 K when increasing *x* from 0.00 to 0.05, respectively. The reported magnetic entropy change for both samples was considerably remarkable and equal to 5.8 J kg^−1^ K^−1^ for *x* = 0.00 and 7.3 J kg^−1^ K^−1^ for *x* = 0.05, respectively, for *μ*_0_*H* = 5 T, confirming that these materials are promising candidates for magnetic refrigeration applications. The mean-field theory was used to study the magnetocaloric effect within the thermodynamics of the model. Satisfactory agreement between experimental data and the mean-field theory has been found.

## Abstract 2

La_0.67−*x*_Bi_*x*_Ba_0.33_MnO_3_ (*x* = 0 and 0.05) ceramics were prepared *via* the sol–gel method.

## Graphical abstract

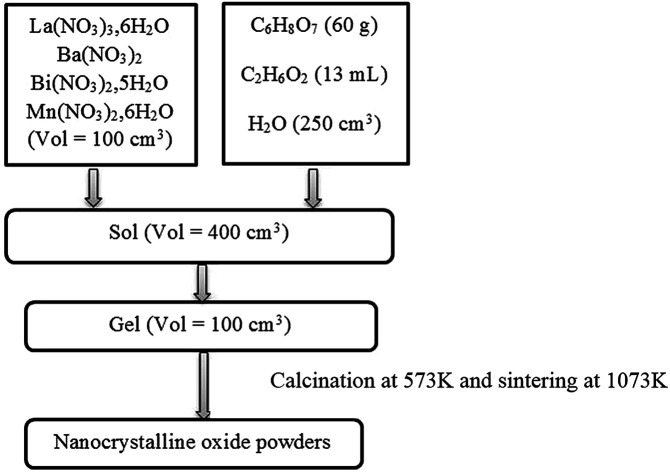

## 1 Introduction

Over the past few years, the perovskite manganites with ABO_3_-type compounds Tr_1−*x*_M_*x*_MnO_3_ (where Tr stands for a trivalent rare-earth element such as Bi^3+^, La^3+^ or Pr^3+^, and M for the divalent alkaline earth ions such as Sr^2+^, Ca^2+^ or Ba^2+^), have been extensively studied due to their extraordinary magnetic and electronic properties as well as their promise for potential technological applications.^1,2^ A prominent feature of the mixed-valence perovskite manganite materials is an insulator–metal (IM) transition accompanied simultaneously by the paramagnetic–ferromagnetic (PM–FM) transition giving rise to a colossal magnetoresistance (CMR) effect.^3^ The existence of the observed CMR near the transition temperature was due to the mixed valence state of Mn, evolving from Mn^3+^ (t^3^_2g_↑e^1^_g_↑, *S* = 2) in the parent atom LaMnO_3_ to Mn^4+^ (t^3^_2g_↑e^0^_g_, *S* = 3/2) to the doped element SrMnO_3_.^4^ The double exchange interaction of the neighboring spin moment of (Mn^3+^, Mn^4+^) coupled through oxygen ions (O^2+^), the small polaron theory and the Jahn–Teller (JT) effect have been proposed to explain the CMR phenomenon near the transition temperature.^5^ In addition, when a field is applied to this material, the unpaired spins are aligned parallel to the field. Since the total entropy of spins plus the lattice remains constant, the magnetic entropy change (−Δ*S*_M_) is removed from the spin system and goes into the lattice, which lowers the magnetic entropy and produces a net heat. On the contrary, when an applied field is removed from a magnetic sample, the spin tends to become random, leading to increment of the entropy and causing the material to cool down. As well known, the maximum of the magnetic entropy change in this kind of material always occurs around its magnetic ordering temperature (*i.e.*, Curie temperature, *T*_C_). Nowadays, there is a need of new advanced magnetics materials with a second order magnetic phase transition, showing a large reversible (−Δ*S*_M_) at low applied fields. Some theoretical works have focused on this subject, for second order phase transition *via* the molecular mean field theory.^6^ For this it is important to know the field dependence of a given magnetic refrigerant sample. The study of the magnetocaloric effect is not only important from the point of view of potential applications; it also provides a tool to understand the intrinsic properties of a material. In Bi based manganites, the lone pair electrons of Bi^3+^ ion hybridize with oxygen 2p orbitals, which in turn reduces the bond length of *d*_Bi–O_ and bond angle of *θ*_Mn–O–Mn_ and increases the bond length of *d*_Mn–O_.^7^

From this viewpoint, this paper reports the structural, magnetic and magnetic entropy change of Bi-substituted perovskite manganites La_0.62_Bi_0.05_Ba_0.33_MnO_3_. It is found that these materials show quite large magnetic entropy changes induced by low magnetic field changes.

## 2 Experimental details

**Fig. 1:**
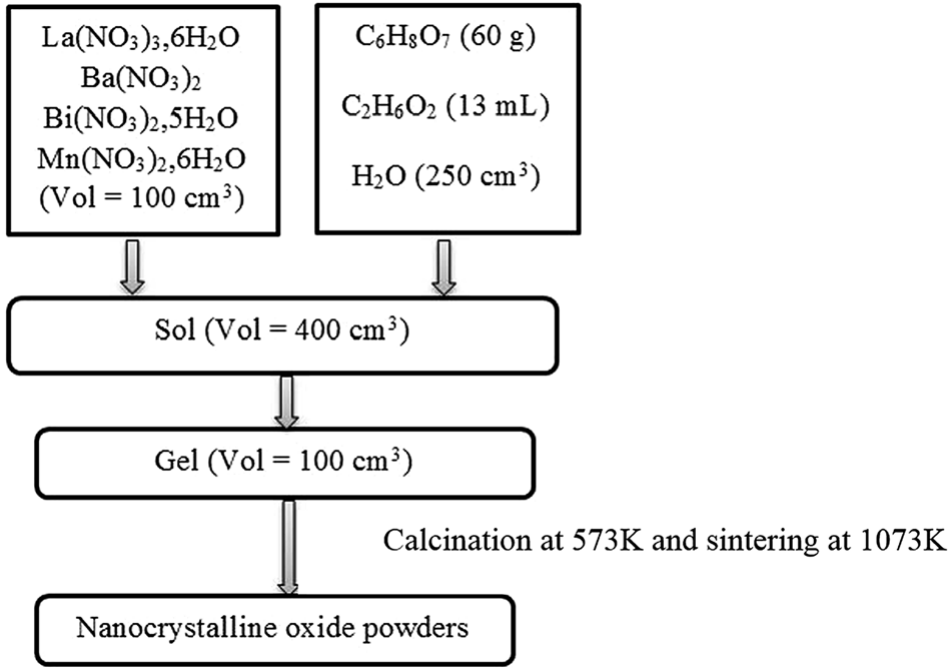
A flow chart illustrating the processing procedure for La_0.67−*x*_Bi_*x*_Ba_0.33_MnO_3_ powders preparation.

Powders of La_0.67−*x*_Bi_*x*_Ba_0.33_MnO_3_ (*x* = 0 and 0.05) were prepared *via* sol–gel route. In this process, La(NO_3_)_3_·6H_2_O, Ba(NO_3_)_2_, Bi(NO_3_)_2_·5H_2_O and Mn(NO_3_)_2_·6H_2_O precursors, all with purity of 99.9%, were weighed in the desired proportions and dissolved with small amounts of water. Ethylene glycol (EG) and citric acid (CA) were used as polymerization/complexation agents, respectively, forming a stable solution. 100 cm^3^ of metallic salts solution was added to 300 cm^3^ of a solution containing a mixture of citric acid (60 g) and ethylene glycol (13 mL). This solution was then heated on a thermal plate under constant stirring, where polymerization occurs in the liquid solution and leads to a homogeneous sol. When the sol is further heated to remove the excess of solvent, an intermediate resin is formed. Calcination of the resin at 573 K in air was performed and sintering at 1073 K for 10 hours. These procedures are outlined in the flow chart of Fig. 1.

The phase purity and structure of sample were identified by X-ray powder diffraction at room temperature using a Siemens D5000 X-ray diffractometer with a graphite monochromatized CuKα radiation (*λ*_CuKα_ = 1.5406 Å) and 20° ≤ 2*θ* ≤ 90° with steps of 0.02° and a counting time of 18 s per step. According to our measurements, this system is able to detect up to a minimum of 3% of impurities. The structure analysis was carried out using the Rietveld method with FULLPROF software (version 0.2-Mars 1998-LLB-JRC).^8^ Scanning electron microscopy (SEM) using a Philips XL30 equipped with a field emission gun at 20 kV was used to characterize La_0.67−*x*_Bi_*x*_Ba_0.33_MnO_3_ morphologies.

Magnetization (*M*) *versus* temperature (*T*) and magnetization *versus* magnetic field (*μ*_0_*H*) were performed by using BS1 and BS2 magnetometers developed in Louis Neel Laboratory at Grenoble. The isothermal curves were determined in the magnetic field range of 0–5 T. The temperature interval is fixed to 2 K in the vicinity of the Curie temperature (*T*_C_). The temperature steps were smaller near *T*_C_ and larger further away.

## 3 Results and discussion

### 3.1 X-ray diffraction and microstructure analysis

**Fig. 2:**
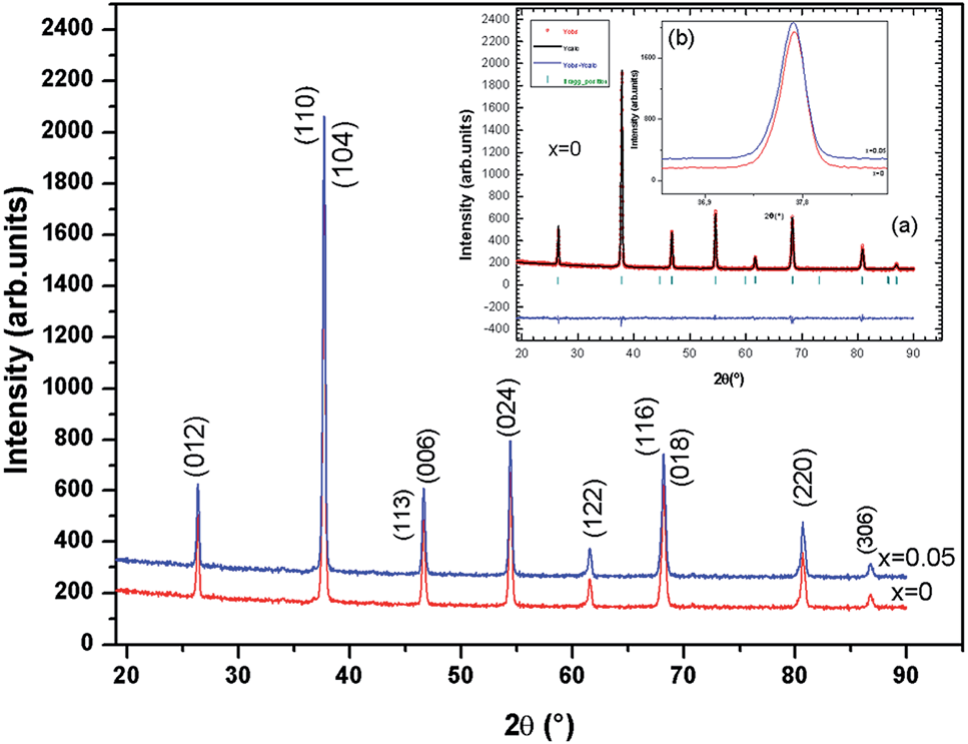
X-ray powder diffraction patterns and Rietveld refinement for the compounds La_0.67−*x*_Bi_*x*_Ba_0.33_MnO_3_ (*x* = 0 and 0.05) at room temperature.

The X-ray diffraction pattern for the samples (*x* = 0.00 and 0.05) is shown in Fig. 2. The samples of La_0.67−*x*_Bi_*x*_Ba_0.33_MnO_3_ are a single phase without detectable secondary phase, within the sensitivity limits of the experiment (a few percent). The Rietveld refinements was successful considering the *R*3̄*c* (no. 167) rhombohedral and centro symmetric space group (inset (a) of Fig. 2, for *x* = 0.0 for example). Standard hexagonal setting of the *R*3̄*c* space group (with *a*_H_ and *c*_H_ cell parameters) was used. The manganite structure (LaAlO_3_ type) is described in this hexagonal setting, with (La/Bi/Ba) atoms at 6a (0, 0, 1/4) position, Mn at 6b (0, 0, 0) and O at 18e (*x*, 0, 1/4) position. This distorted manganite is characterized by *ā ā ā* antiphase oxygen octahedral tilt system (Glazer notation^9^) corresponding to rotations along the three pseudo cubic directions of the manganite. Detailed results of the structural refinements are regrouped in Table 1. It can be observed from the inset (b) in Fig. 2 that the position of the most intense peak shows a slight shift towards low angles with the increase of Bi, indicating that the cell volume of the La_0.67−*x*_Bi_*x*_Ba_0.33_MnO_3_ specimens increases.

**Table 1:** Physical properties of the sample La_1−*x*_Bi_*x*_Ba_0.33_MnO_3_ system, prepared by sol–gel method at room temperature^a^

Crystallographic data			Refinement conditions				Average crystallite size		Strain *ε* (%)	Average grain size *D* (nm)
Lattice parameters		Cell volume	Discrepancy factors				Debye–Scherrer technique *D*_s_ (nm)	Williamson–Hall technique *D*_w_ (nm)		
*a* = *b* (Å)	*c* (Å)	*V* (Å^3^)	*R*_wp_ %	*R*_p_ %	*R*_F_ %	*χ* ^2^ %				

***x* = 0**										
5.5160 (3)	13.5023(1)	355.78(2)	4.12	3.43	2.78	1.85	38	49	0.16	220

***x* = 0.05**										
5.50184 (2)	13.5141(4)	356.40(1)	5.23	3.42	3.42	1.25	35	40	0.14	260

a *a* and *c*: hexagonal cell parameters; *V*: cell volume; *R*_wp_, *R*_p_; *R*_F_: the residuals for, respectively, the weighted pattern, the pattern and the Bragg structure factor; *χ*^2^: the goodness of fit. The numbers in parentheses are estimated standard deviations to the last significant digit.

In order to quantitatively discuss the ionic match between A and B sites in perovskite compounds, a geometrical quantity, noted Goldschmidt tolerance factor (*t*), is usually introduced and is defined as:^10^
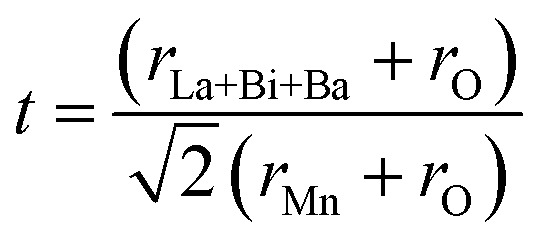
Here *r*_La+Bi+Ba_; *r*_Mn_ and *r*_O_ are the average ionic radii of A, B and oxygen, respectively in the perovskite ABO_3_ structure. Manganite compounds have a perovskite structure if their tolerance factor lies in the limits of 0.75 < *t* < 1 and in an ideal case when the value must be equal to unity. In the present work, the tolerance factor of La_0.62_Bi_0.05_Ba_0.33_MnO_3_ is calculated from Shannon's ionic radii (*r*_La_^3+^ = 1.22 Å, *r*_Bi_^3+^ = 1.24 Å, *r*_Ba_^2+^ = 1.47 Å, *r*_Mn_^3+^ = 0.645 Å, *r*_Mn_^4+^ = 0.53 Å, *r*_O_^2−^ = 1.35 Å)^11,12^ and it is found to be *t* = 0.9595 and 0.9599 for *x* = 0 and *x* = 0.05, respectively, which is within the range of stable perovskite structure.

The value of average crystallite size was estimated from the full width at half maximum (FWHM) of X-ray diffraction peaks. The effects of synthesis, instruments and processing conditions were taken into consideration while making the calculation of crystallite size. The dependence of the size effect is given by Scherrer's formula: 
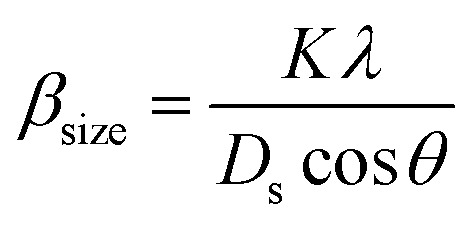
, where *λ* is the wavelength of CuKα radiation (*λ* = 1.5406 Å), *K* is grain shape factor (=0.89) and *D*_s_ is the thickness of the crystal. Using the Williamson–Hall (W–H) method,^13^ the average values of both *D*_w_ and lattice strain (*ε*) can be obtained from the intercept and the slope of the following relation, respectively,
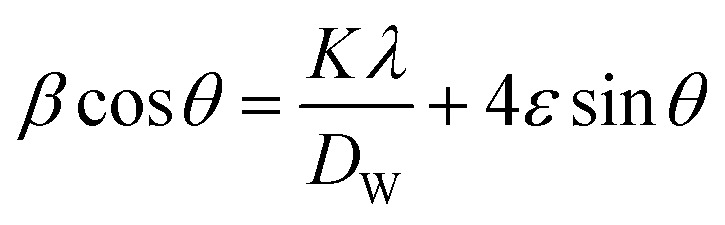
where *β* is the full-width at half-maximum of an XRD peak, *θ* is the Bragg angle, and *K* = 0.9 is the shape factor. The values of average crystallite size *D*_S_, *D*_W_ and micro-strain of La_0.67−*x*_Bi_*x*_Ba_0.33_MnO_3_ compounds are tabulated in Table 1. The particle size, calculated in the present system using Williamson–Hall technique, is larger than the particle size obtained from Debye–Scherrer method because the broadening effect due to strain is completely excluded in Debye–Scherrer technique.^14^

**Fig. 3:**
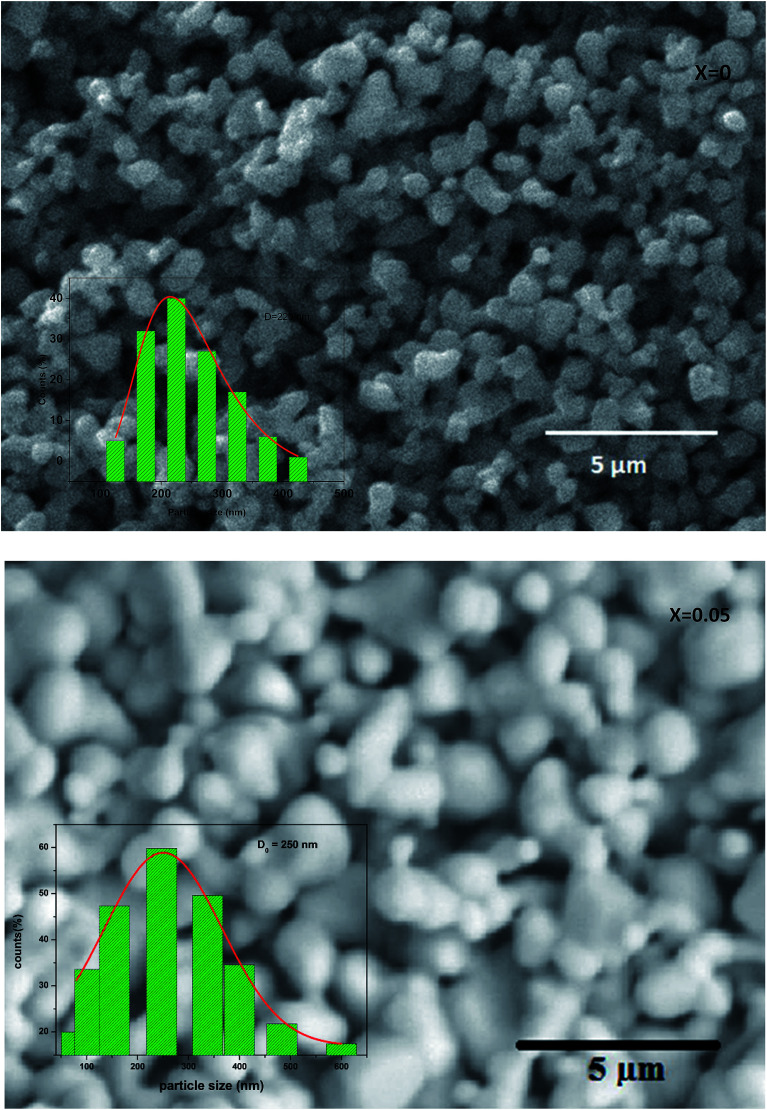
Scanning electron micrograph for La_0.67−*x*_Bi_*x*_Ba_0.33_MnO_3_ (*x* = 0 and 0.05) manganite. The inset: dispersion histogram.

Fig. 3 shows the SEM photograph of the compounds. The samples contained connected particles with hexagonal shape and clear grain boundaries. These particles are largely agglomerated with a broad size distribution. The average value of thickness of both compounds is listed in Table 1.

After measuring the diameters of all the particles in SEM image, the size distribution histogram is fitted with the log-normal function expressed as:
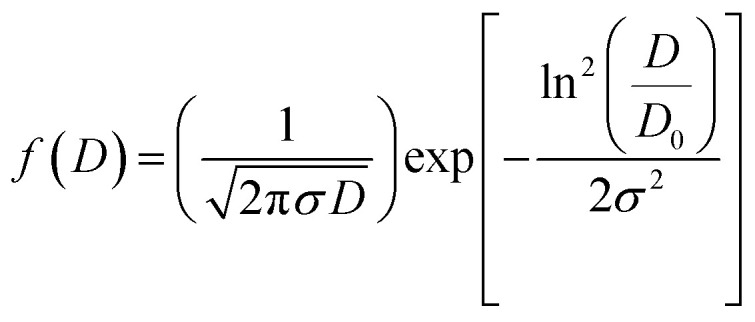
Here *D*_0_ and *σ* are the average diameter obtained from the SEM results and the data dispersions, respectively. The inset of Fig. 3 shows the dispersion histogram. The mean diameter 〈*D*〉 and standard deviation *σ*_D_ were determined using these relations:^15,16^〈*D*〉 = *D*_0_ exp(*σ*^2^/2)*σ*_D_ = 〈*D*〉[exp(σ^2^) − 1]^1/2^

The results analysis showed 〈*D*〉 ≥ 397.48 μm and *σ*_D_ = 291.53 μm.

### 3.2 Bulk magnetization

**Fig. 4:**
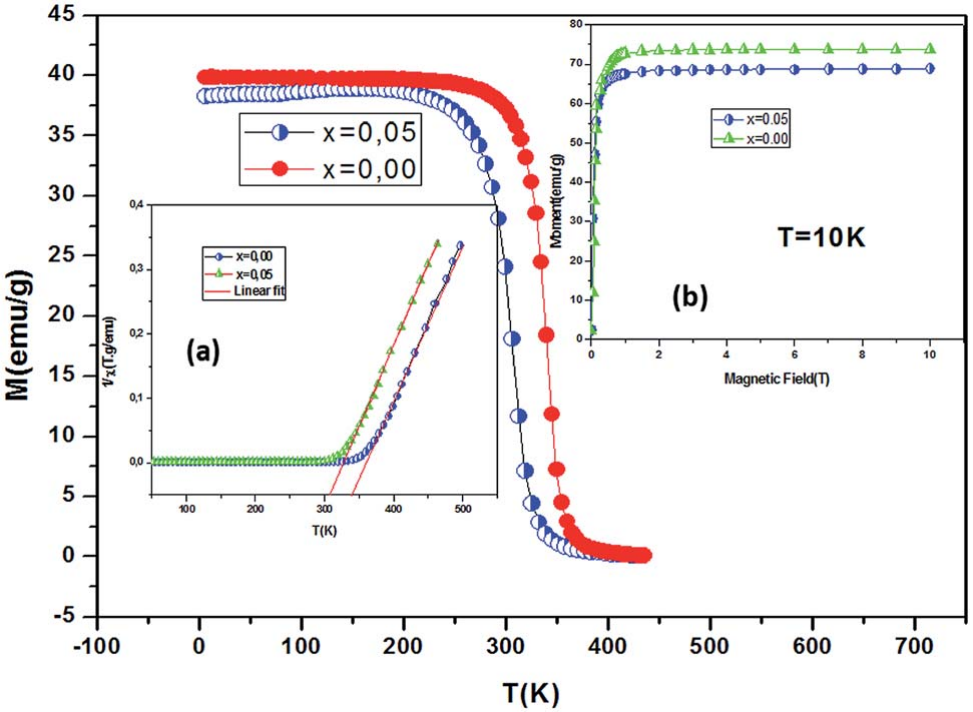
Temperature dependence of the magnetization measured at *μ*_0_*H* = 0.05 T for La_0.67−*x*_Bi_*x*_Ba_0.33_MnO_3_ sample (*x* = 0 and 0.05). Inset (a) show temperature dependence of the inverse dc magnetic susceptibility from magnetization measurements. Inset (b) variation of the magnetization as a function of applied magnetic field at 10 K.

Low-field magnetization (*M*) *versus* temperature was first measured for the samples, in order to have an estimation of the transition temperature (*T*_C_). The result is presented in Fig. 4. The *M*(*T*) curve reveals that when increasing temperature, the samples exhibit a magnetic transition from paramagnetic (PM) state to ferromagnetic (FM) one. This transition occurs at the Curie temperature (*T*_C_) which is obtained from the peak of d*M*/d*T* curve. The Curie temperature decreases from 340 K to 306 K when increase *x* from 0.00 to 0.05, respectively for *μ*_0_*H* = 0.05 T. The Curie temperature of the Bi-doped compound was found to be lower than that of the undoped sample. This indicates that Bi substitution appears to weaken the magnetic interaction in the sample. Theoretical calculation has shown that off-center shifts of the ions with *n*s^2^ electronic configuration results in the structural distortion and minimization of the Coulombic energy.^17^ An orientation of the 6s^2^ lone pair toward a surrounding anion (O-2p) can produce a local distortion or even hybridization between Bi-6s-orbitals and O-2p orbitals,^18^ leading to the block of the movement of e_g_ electrons through the Mn–O– Mn bridges (stronger localization).

The inset of Fig. 4 shows the temperature dependence of the inverse magnetic susceptibility of *x* = 0 and *x* = 0.05. It could be fitted to the Curie–Weiss law just above *T*_C_ (the PM region): *χ* = *C*/*T* − *θ*_CW_, where *θ*_CW_ is the Weiss temperature and *C* is the Curie constant defined as: 
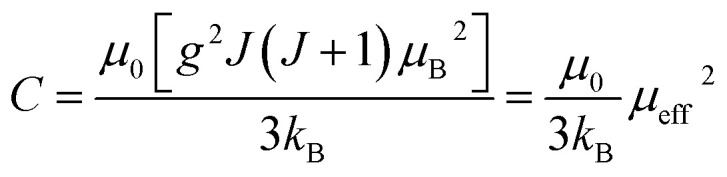
, where *μ*_0_ = 10^7^ H m^−1^ is the permeability, *g* is the Landé factor, *μ*_B_ = 9.27 × 10^24^ J T^−1^ is the Bohr magneton, *k*_B_ = 1.38 × 10^23^ J K^−1^ is the Boltzmann constant, *J* = *L* + *S* is the total moment and *μ*_eff_ is the effective paramagnetic moment. We can determine the effect of paramagnetic moment (*μ*^exp^_eff_) from the curie constant. The theoretical *μ*^calc^_eff_ is estimated using the following expression: 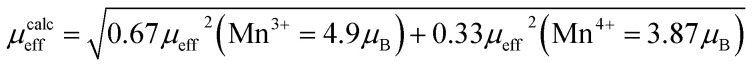. The parameters *μ*^exp^_eff_ and *μ*^calc^_eff_ are summarized in Table 2. It is found that the *μ*^exp^_eff_ is greater as compared to *μ*^calc^_eff_. This discrepancy validates the formation of ferromagnetic spin clusters within the paramagnetic state.^19^ A linear fit yields positive Curie–Weiss temperature *θ*_CW_ = 312 K (*x* = 0.05). This result confirms a mean FM interaction between spins for all samples (Table 2). Moreover, this value is higher than *T*_C_, which may be due to the existence of short range FM ordering.^19^

**Table 2:** Transition temperature *T*_C_, *W*/*W*_0_ and Curie Weiss temperature *θ*_W_ as a function of *x* content for La_1−*x*_Bi_*x*_Ba_0.33_MnO_3_ samples

*x*	*T*_C_ (K)	*W*/*W*_0_ (10^−2^)	*θ*_CW_ (K)	*θ*_Mn–O–Mn_ (°)	*d*_Mn–O_ (Å)	*μ* ^exp^_eff_ (*μ*_B_)	*μ* ^calc^_eff_ (*μ*_B_)
0	340	4.73	348	165.32	1.959	5.32	4.586
0.05	306	4.66	312	165.12	1.965	5.81	4.586

The structure analysis shows that the unit cell becomes slightly larger as the 6s^2^ lone pair character becomes dominant, it has been shown that the Bi–O bond is shorter than the La–O, despite of the similar ionic radius of La^3+^ and Bi^3+^ ions.^20^ This can be interpreted as arising from the rather covalent character of the Bi–O bonds. The electronegativity of Bi enhances hybridisation between 6s^2^ of Bi^3+^ orbitals and 2p of O^2−^ orbitals and this hybridisation produces a local distortion. It is observed that transition temperature *T*_C_ decreases with increase in Bi ratio. This is presumably due to tilts the MnO_6_ octahedra, resulting in a reduced overlap between the Mn-3d and O-2p orbitals.^21^ It should also be noted that the La_0.67_Ba_0.33_MnO_3_ sample is ferromagnetic while Bi_0.67_Ba_0.33_MnO_3_ is antiferromagnetic, indicating a competition between the double exchange and the antiferromagnetic super exchange in these compounds can decrease *T*_C_. This phenomenon has been observed in the compound Bi_0.6−*x*_La_*x*_Ca_0.4_MnO_3_.^22^

### 3.3 Effect of Bi on magnetocaloric properties

The change of magnetic entropy of magnetic compounds has the largest value near a phase transition. According to the classical thermodynamic theory, the isothermal magnetic entropy change (–Δ*S*_M_) produced by the variation of a magnetic field from zero to *μ*_0_*H*_ma*x*_ is given by:^23,24^
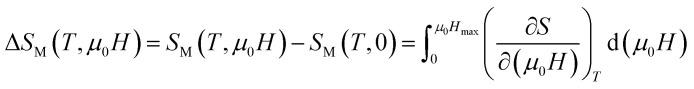


**Fig. 5:**
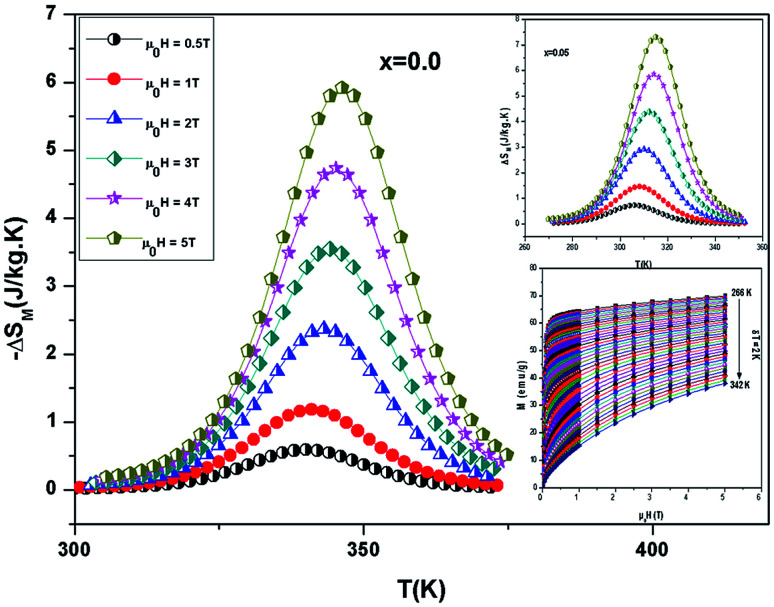
Temperature dependence of magnetic entropy change under different external fields for La_0.67−*x*_Bi_*x*_Ba_0.33_MnO_3_ samples. Inset: magnetization *versus* applied magnetic field *μ*_0_*H*, measured at different temperatures, for example the sample La_0.62_Bi_0.05_Ba_0.33_MnO_3_.

The magnetic entropy is related to the magnetization *M*, magnetic field strength *μ*_0_*H* and absolute temperature *T* through the Maxwell relation:
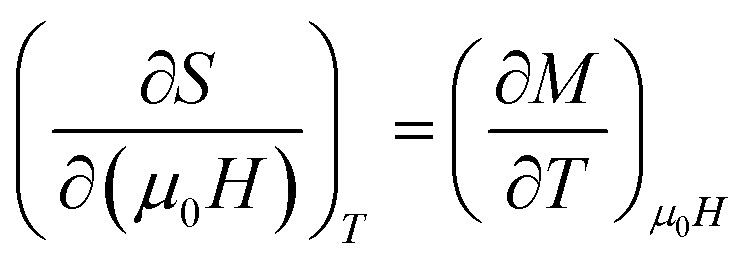


In the case of magnetization measurement in small discrete magnetic fields and temperature interval Δ*T*, Δ*S*_M_ can be approximated to:
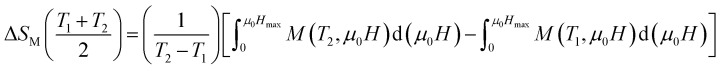


The −Δ*S*_M_(*T*) data calculated from the *M*(*μ*_0_*H*) curves (inset in Fig. 5) at different magnetic fields for the La_0.67−*x*_Bi_*x*_Ba_0.33_MnO_3_ (*x* = 0 and 0.05) are plotted in Fig. 5. The compounds exhibit large changes in magnetic entropy around Curie temperature (*T*_C_), which is a characteristic property of simple ferromagnets due to the efficient ordering of magnetic spins at the temperature induced by magnetic field.^25^ Large magnetic entropy changes Δ*S*^max^_M_ are reported for all the samples and are summarized in Table 3. The magnitude of (−Δ*S*^max^_M_(*T*)) for all samples increases with increasing the applied magnetic field (inset of Fig. 5). For example, the maximum magnetic-entropy value increases from 2.37 J kg^−1^ K^−1^ for *x* = 0.00 to 2.8 J kg^−1^ K^−1^ (2*T*) and 5.8 J kg^−1^ K^−1^ for *x* = 0.00 to 7.3 J kg^−1^ K^−1^ for *x* = 0.05 respectively (5*T*). Guo *et al.*^26^ indicated that the large magnetic entropy change in perovskite compounds could originate from the spin–lattice coupling in the magnetic ordering process. Strong coupling between spin and lattice is corroborated by the observed significant lattice change accompanying magnetic transition in perovskite manganites.^27^ The lattice structural change in the Mn–O bond distance as well as in the 〈Mn–O–Mn〉 bond angle would in turn favor the spin ordering. Thus a more abrupt variation of magnetization near Curie temperature (*T*_C_) occurs, resulting in a large magnetic entropy change as a large MCE.

**Table 3:** Δ*S*_M_ and RCP values for the present samples and for some previous works

Composition	*T*_C_ (K)	(−Δ*S*^max^_M_) (J kg^−1^ K^−1^)	RCP (J kg^−1^)	*μ*_0_ *H* (T)	Ref.
Gd_5_(Sr_2_Ge_2_)	275	18.5	535	5	24
Gd	294	10.2	410	5	25
La_0.5_Sm_0.1_Sr_0.4_Mn_0.95_In_0.05_O_3_	308	4.50	193.48	5	26
La_0.67_Sr_0.33_Mn_0.9_Cr_0.1_O_3_	328	5	—	5	27
La_0.67_Ba_0.33_MnO_3_	346	5.80	151	5	This work
La_0.62_Bi_0.05_Ba_0.33_MnO_3_	310	7.30	209	5	This work
Gd	297	4	120	2	28
MnFeP_0.45_As_0.5_	300	14.5	188	2	28
La_0.7_Sr_0.3_Mn_0.95_Ti_0.05_O_3_	308	2.2	90	2	29
La_0.7_Sr_0.3_Mn_0.9_Fe_0.1_O_3_	260	1.7	83	2	30
La_0.67_Ba_0.33_MnO_3_	343	2.37	39	2	This work
La_0.62_Bi_0.05_Ba_0.33_MnO_3_	308	2.80	80	2	This work

**Fig. 6:**
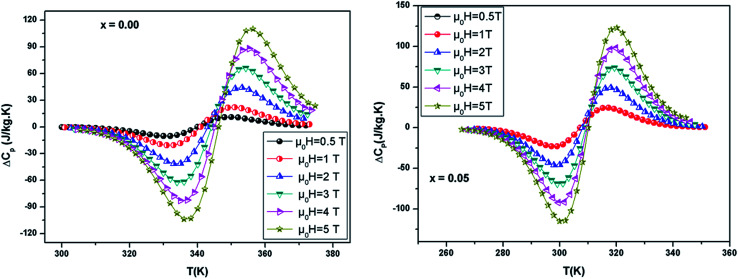
Change of specific heat of the samples as a function of temperature at different magnetic field.

The change of magnetic entropy can be also calculated from the field dependence of the specific heat by the following integration:
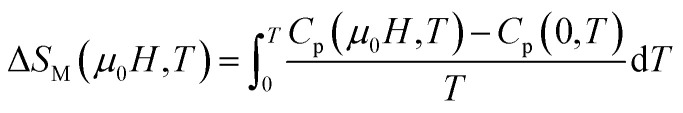


From this equation, it determine the change of specific heat induced by the external magnetic field zero to *μ*_0_*H* as: 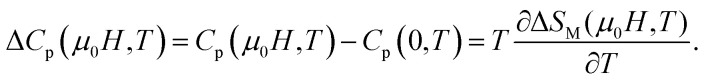


Fig. 6 shows the temperature dependence of Δ*C*_p_(*μ*_0_*H*, *T*) under different field variations for the samples (for example *x* = 0.05) calculated from the Δ*S*_M_(*μ*_0_*H*, *T*). The Δ*C*_p_(*μ*_0_*H*, *T*) undergoes a sudden change from positive to negative around *T*_C_ with a positive value above *T*_C_ and a negative value below *T*_C_. The maximum/minimum value of Δ*C*_p_(*μ*_0_*H*, *T*) observed at 320/300 K, exhibits an increasing trend with applied field and is obtained to be 122.4/−115.43 J kg^−1^ K^−1^ for *x* = 0.05 at 5 T.

**Fig. 7:**
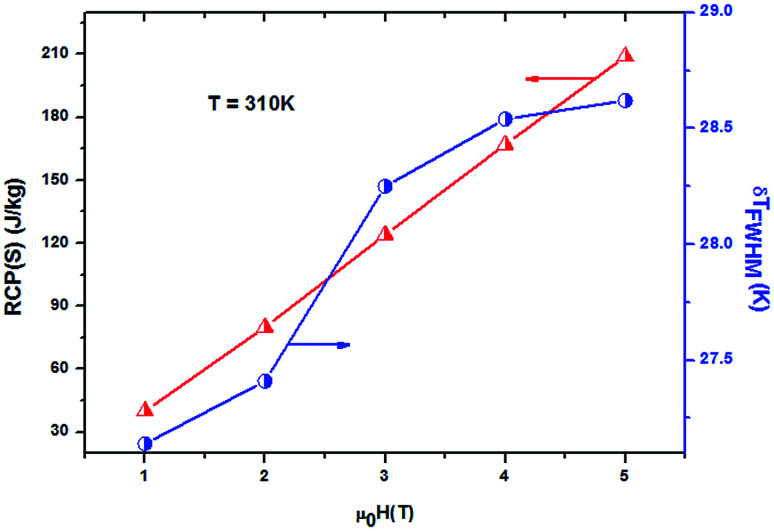
Magnetic field dependence of RCP and *δT*_FWHM_ at 310 K for *x* = 0.05 for example.

It should be noted that (−Δ*S*^max^_M_) is not the only parameter deciding about an applicability of material. To estimate if a material can be a good candidate for magnetic refrigeration (MR), Gschneidner and Pecharsky^28^ defined the relative cooling power (RCPS), which is the important index which is used to evaluate the cooling efficiency of a magnetic refrigerant. It is defined as the product between the maximum values of the magnetic entropy change (−Δ*S*^max^_M_) and the full width at half maximum *δT*_FWHM_ of the magnetic entropy change curve (RCP(S) = −Δ*S*^max^_M_ × *δT*_FWHM_).^29^ This parameter corresponds to the amount of heat that can be transferred between the cold and hot parts of the refrigerator in one ideal thermodynamic cycle. The results are summarized in Table 3. Fig. 7 shows the absolute value of RCPS and *δT*_FWHM_ for the sample (*x* = 0.05) *versus* applied field at 310 K. It can be seen from this figure that RCPS (*δT*_FWHM_) increases monotonically as the field increases. The value of RCP is about 51% of Gd at 294 K for *μ*_0_*H* = 5 T.^30^ To evaluate the applicability of ours samples as a magnetic refrigerant, the obtained values of Δ*S*_M_ in our study are compared in Table 3 with those reported in the literature for several other magnetic compounds.^31–37^

### 3.4 Modeling the magnetic properties

The Weiss molecular mean field model is a standard model in magnetism. Because of its simplicity, this model is still used in current research for a wide range of magnetic materials, although its limitations are well known. This concept of a molecular field assumes that the magnetic interaction between magnetic moments is equivalent to the existence of an exchange interaction depending on the magnetization *M*:^38^*H*_eff_ = *H* + *H*_exch_ = *H* + *λM*where *H* and *H*_exch_ are the external magnetic field, the exchange magnetic field and *λ* the mean field exchange parameter respectively.

Amaral *et al.* proposed a model based on mean field theory and presented an approach of applying this method scenario to isotherm magnetization *M*(*T*, *H*) measurements.^39^ In our study, it consider the general mean field law:^40^*M*(*H*, *T*) = *B*_J_[(*H* + *H*_exch_)/*T*], the Brillouin function *B*_J_ is written as: 
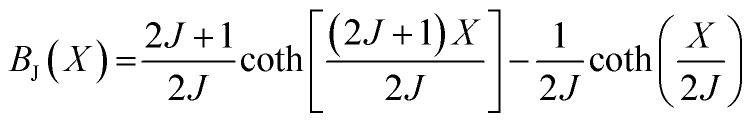
, where 
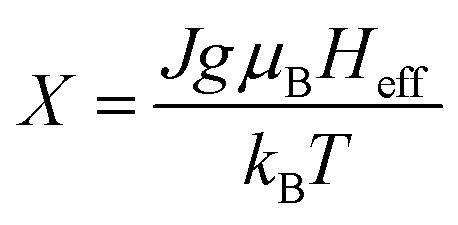
, *J* is the total angular momentum in the lattice, *g* is the gyromagnetic factor (landé factor), *μ*_B_ is the Bohr magnetron and *k*_B_ is the Boltzmann's constant. The mean field exchange parameter *λ* is not predetermined. Then for corresponding values with the same (*H* + *H*_exch_)/*T*, *M* is also the same, the value of the inverse *B*_J_^−1^(*M*) function,^41^
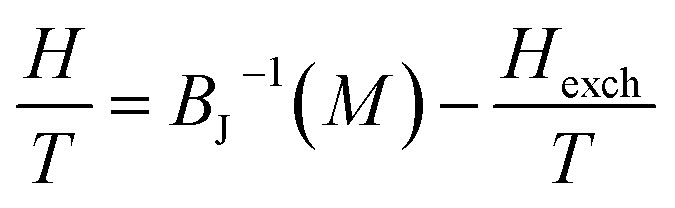


**Fig. 8:**
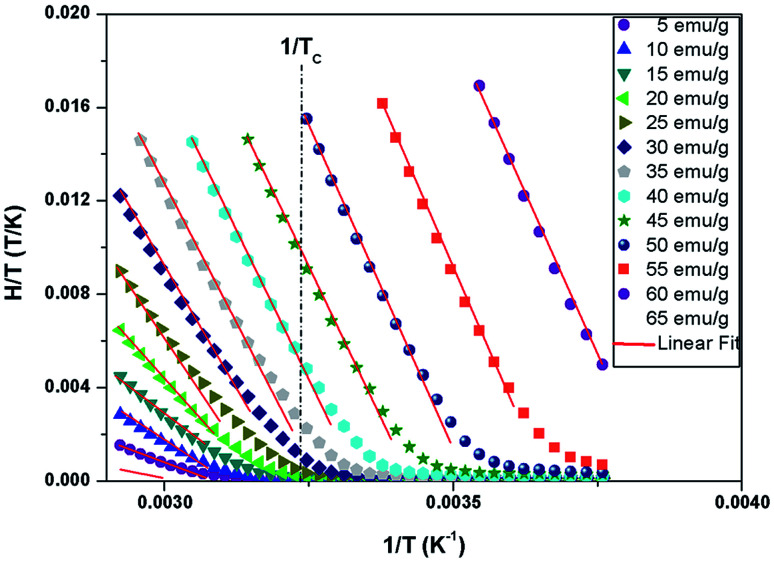
*H*/*T versus* 1/*T* curves with constant values of magnetization per curve for La_0.62_Bi_0.05_Ba_0.33_MnO_3_ compound.

The study of the exchange field induced by the magnetization change makes it possible to find the value of the average field exchange parameter *λ*. Fig. 8 shows *H*/*T versus* 1/*T* for some of the values of *M* (5 emu per g per step) from 266 K to 342 K for *x* = 0.05. According to the mean scaling method such *H*/*T versus* 1/*T* curves should show a series of straight lines at different temperature. The linear relationship between *H*/*T* and 1/*T* is kept. Linear fits are then easily made to each isomagnetic line. Typically, the interpolation step was of 1 emu g^−1^. The slope of this isomagnetic line, will then give the exchange field (*H*_exch_).

For all compounds in the paramagnetic domain or the materials of domain ordered such as anti-ferromagnetic, it can always expand increasing *M* in powers of *H*, or *H* in powers of *M*. In this latter approach it stop at the third order and considering that the magnetization is an odd function of field, it can write:^42^*H*_exch_ = *λ*_1_*M* + *λ*_3_*M*^3^

**Fig. 9:**
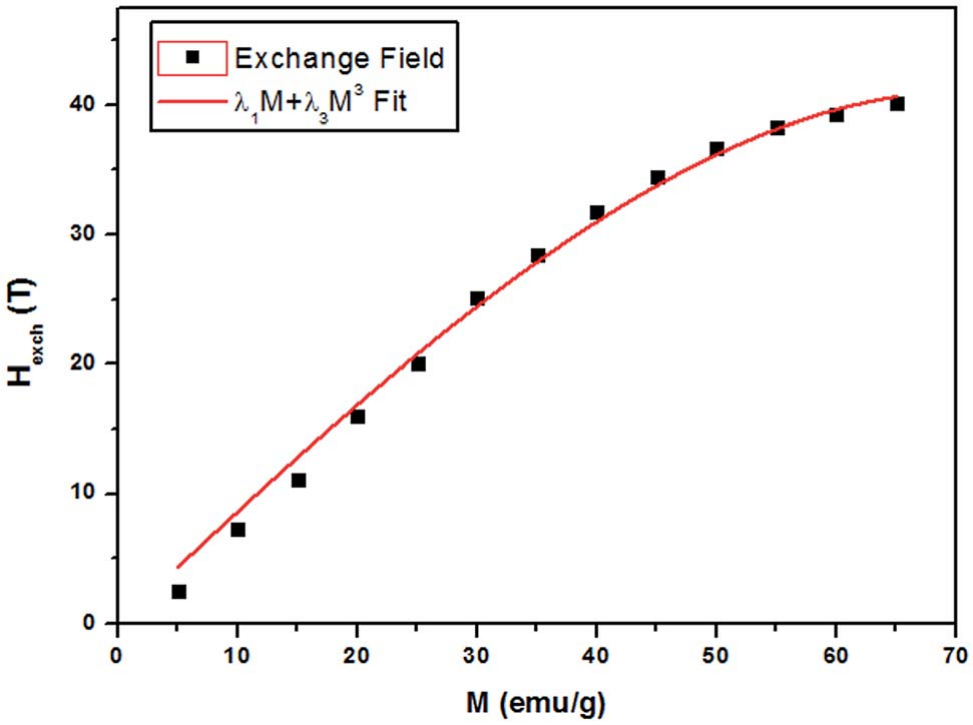
Exchange field *versus* magnetization for La_0.62_Bi_0.05_Ba_0.33_MnO_3_ sample, with the function *λ*_1_*M* + *λ*_3_*M*^3^ fit.

**Fig. 10:**
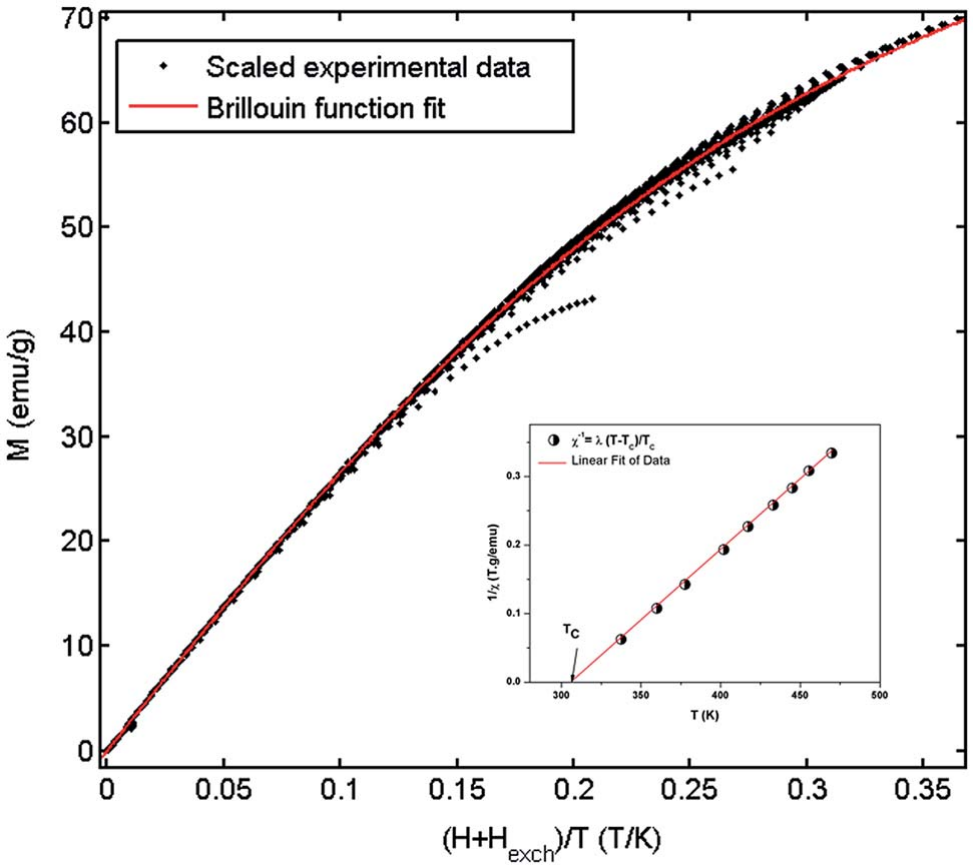
Mean-field scaling plot and Brillouin function fit. Inset 1/*χ versus T* of La_0.62_Bi_0.05_Ba_0.33_MnO_3_ compound.

Fig. 9 shows the evolution of the exchange field *versus* the magnetization for the La_0.67−*x*_Bi_*x*_Ba_0.33_MnO_3_ (*x* = 0.05 for example). The experimental data should be included for the fit by eqn (*H*_exch_ = *λ*_1_*M* + *λ*_3_*M*^3^). The results show a very small dependence on *M*^3^ (*λ*_3_ = −1.3984 × 10^−5^ (T g emu^−1^)^3^), is found for this second order transition system, thus *H*_exch_ = *λ*_1_*M* with *λ*_1_ = 1.25 T g emu^−1^. After obtaining the mean field exchange parameter the next step of this method consists on building the scaling plot of *M vs.* (*H* + *H*_exch_)/*T* (Fig. 10). It has successfully fitted the scaled magnetization data with the Brillouin function. From the scaling plot and the subsequent fit with the saturation magnetization equal to 72 emu g^−1^ (this value is close to the experimental one (*M*_s_ = 69 emu g^−1^ at 10 K) (inset b of Fig. 4), and the value of the total angular momentum of the manganite is *J* = 1.9.

The magnetization measurements in the law temperature range show that the saturation magnetization is about *M*^theor^_s_ = *Jgμ*_B_ = 3.67*μ*_B_/f.u. This value is close to the experimental value *M*^exper^_s_ = 3.7*μ*_B_ at 10 K. The value of *M*^theor^_s_ per formula unit is given by: *M*^theor^_s_ = (*n*_Mn^3+^_)*M*_sMn^3+^_ + (*n*_Mn^4+^_)*M*_sMn^4+^_, where *M*_sMn^3+^_ = 4*μ*_B_ and *M*_sMn^4+^_ = 3*μ*_B_ are the magnetic moments, *n*_Mn^3+^_ = 0.67 and *n*_Mn^4+^_ = 0.33 are the contents of Mn^3+^ and Mn^4+^ ions respectively. It deduce that the total angular momentum of the compound is *J* = 1.835 (it was assumed that *g* = 2).

**Fig. 11:**
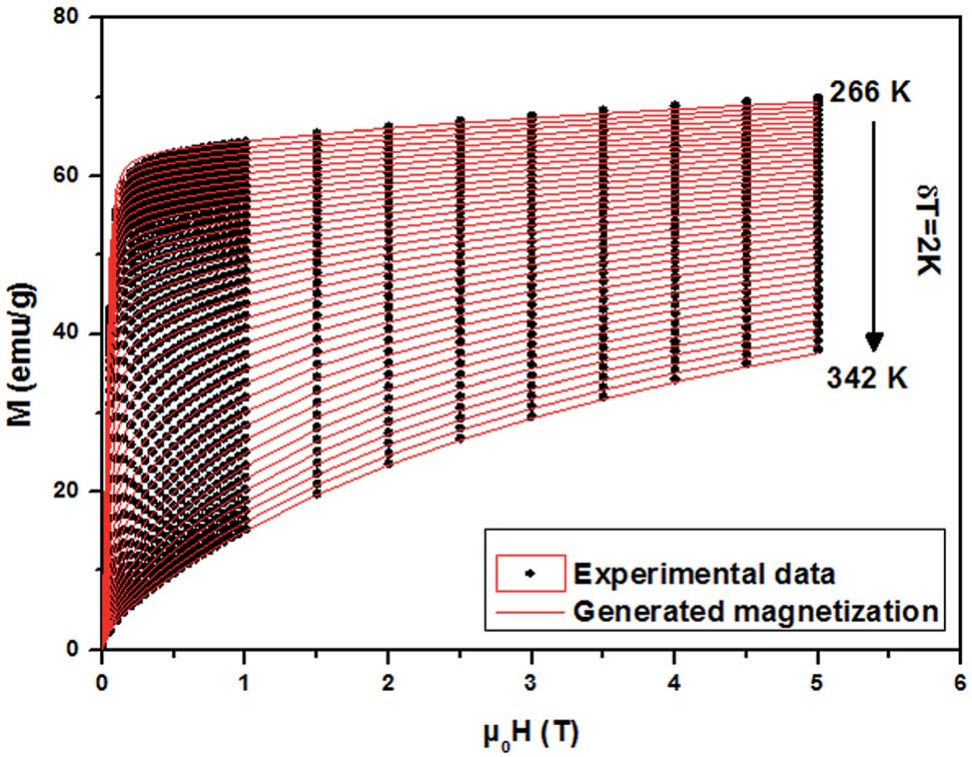
Experimental magnetization *versus μ*_0_*H* (black symbols) of La_0.62_Bi_0.05_Ba_0.33_MnO_3_ sample and the interpolation using the mean field method (red lines).

From a linear approximation of the susceptibility 
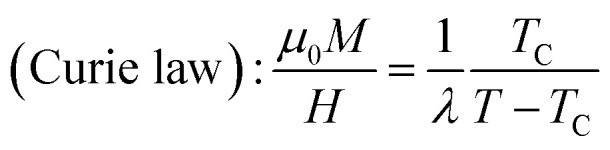
. Inset of the Fig. 10 shows the evolution of the inverse susceptibility (1/*χ*) *versus* temperature for the La_0.62_Bi_0.05_Ba_0.33_MnO_3_ compound. The straight line represents the Curie–Weiss law with *λ* = 1.25 T g emu^−1^ and *T*_C_ = 306 K. The intersection is obtained at a temperature value equals to the critical one. At this point, the susceptibility becomes infinite, which corresponds to the ferromagnetic–paramagnetic transition. Fig. 11 shows how experimental data can be described using the mean-field method. A good agreement between the experimental *M*(*T*, *μ*_0_*H*) curves and the mean-field generated curves with the obtained parameters, except near the paramagnetic-ferromagnetic transition (*T*_C_), which are not adequately described. This result is probably due to the formation of magnetic domains and critical effects.

### 3.5 Estimating the magnetocaloric effect (MCE)

**Fig. 12:**
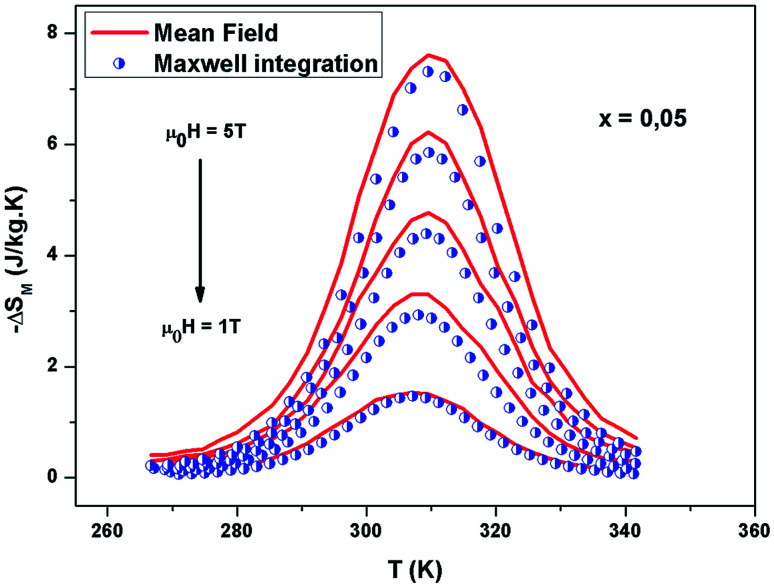
Experimental and calculated magnetic entropy as a function of temperature for *x* = 0.05.

The mean field approach allows us to estimate magnetic entropy variation Δ*S*_M_ within the thermodynamics of the model and without using the usual numerical integration of a Maxwell relation.^43^ Within the mean field approach, Δ*S*_M_ between magnetic fields *H*_1_ and *H*_2_ can be calculated using the following general expression, which also accounts for a possible dependence of *λ* on *T*:
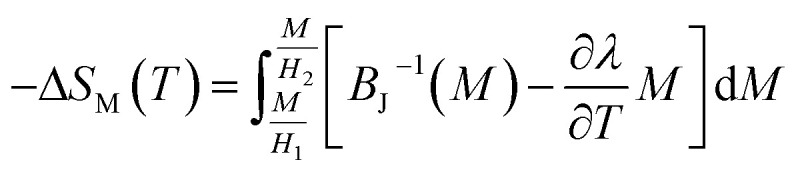


The linearity relation between *H*/*T* and 1/*T* in Fig. 8 improves that the mean field exchange parameter *λ* is independent of the temperature *T*. So the following equation will be simplified:
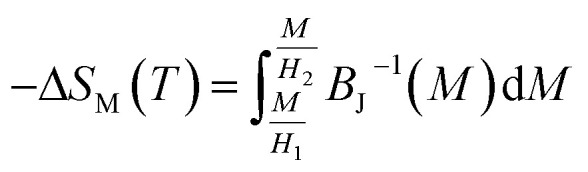


Fig. 12 shows the evolution of the magnetic entropy change (−Δ*S*_M_) data as a function of temperature at several magnetic applied fields for the La_0.62_Bi_0.05_Ba_0.33_MnO_3_ compound, by using the Maxwell relation and that basing on mean field theory. Both results are in good agreement, except close to *T*_C_, here an excepted small difference appears, due to the formation of magnetic domains and critical effects.

## 4 Conclusion

In summary, single phase La_0.67−*x*_Bi_*x*_Ba_0.33_MnO_3_ (*x* = 0.00 and 0.05) compounds were prepared by the sol–gel technic. Using the magnetization measurements, magnetic and magnetocaloric effect have been studied. These compounds show second order ferromagnetic–paramagnetic phase transition, with a large magnetic entropy change. It have studied the mean-field scaling method for these samples. The insight that can be gained from the use of this methodology for a given magnetic system can be of great interest. In a simplistic approach, it can say that if this scaling method does not follow a molecular mean-field behavior, other methods must be pursued in order to interpret the magnetic behavior of the system. The mean-field scaling method is able to determine the exchange parameters *J*, *λ* and *g* of ours samples. These factors allow estimating some magnetic properties.

## Conflicts of interest

There are no conflicts to declare.

## Supplementary Material

## References

[cit1] Khlifi M., Dhahri E., Hlil E. K. (2014). J. Alloys Compd..

[cit2] Wang G. F., Li L. R., Zhao Z. R., Yu X. Q., Zhang X. F. (2014). Ceram. Int..

[cit3] Dagotto E., Hotta T., Moreo A. (2001). Phys. Rep..

[cit4] Dhahri A., Dhahri E., Hlil E. K. (2017). J. Alloys Compd..

[cit5] Von Helmolt R., Wecker J., Holzapfel B., Schultz L., Samwer K. (1993). Phys. Rev. Lett..

[cit6] Bean C. P., Rodbell D. S. (1962). Phys. Rev..

[cit7] Lalitha G., Venugopal Reddy P. (2010). J. Alloys Compd..

[cit8] Rodriguez Carvajal J. , FULLPROF, Laboratoire Leon Brillouin (CEACNRS), 2000–2005

[cit9] Glazer A. M. (1972). Acta Crystallogr..

[cit10] Goldschmidt V. M. , Geochemistry, Oxford University Press, 1958, p. 730

[cit11] Shannon R. D. (1976). Acta Crystallogr..

[cit12] Lalitha G., Venugopal Reddy P. (2010). J. Alloys Compd..

[cit13] Williamson G. K., Hall W. H. (1953). Acta Metall..

[cit14] Mohamed A. G., Ghodhbane S., Dhahri A., Dhahri J., Hlil E. K. (2016). J. Alloys Compd..

[cit15] Aslibeiki B., Kameli P., Ehsani M. H. (2016). Ceram. Int..

[cit16] Lavorato C., Lima Jr E., Tobia D., Fiorani D., Troiani H. E., Zysler R. D., Winkler E. L. (2014). Nanotechnology.

[cit17] Sugawara F., Iida S., Syono Y., Akimoto S. (1968). J. Phys. Soc. Jpn..

[cit18] Hill N. A., Rabe K. M. (1999). Phys. Rev. B: Condens. Matter Mater. Phys..

[cit19] Dhahri A., Jemmali M., Taibi K., Dhahri E., Hlil E. K. (2015). J. Alloys Compd..

[cit20] Barandiarana M., Gutierrez J., Righi L., Amboage M., Pena A., Hernandez T., Insausti M., Rojo T. (2001). Physica B.

[cit21] Colossal magneto-resistance oxides, ed. Y. Tokura, Gordon and Breach, London, 2000

[cit22] Zhang R. R., Kuang G. L., Yin L. H., Sun Y. P. (2010). J. Appl. Phys..

[cit23] Dhahri M., Zaidi A., Cherif K., Dhahri J., Hlil E. K. (2017). J. Alloys Compd..

[cit24] Dhahri Ah., Dhahri E., Hlil E. K. (2014). Appl. Phys. A.

[cit25] Bhattacharyya A., Chatterjee S., Giri S., Majumdar S. (2009). Eur. Phys. J. B.

[cit26] Guo Z. B., Du Y. M., Zhu J. S., Huang H., Ding W. P., Feng D. (1997). Phys. Rev. Lett..

[cit27] Radaelli P. G., Cox D. E., Marezio M., Cheong S. W., Schiffer P. E., Ramirez A. P. (1995). Phys. Rev. Lett..

[cit28] Pecharsky V. K., Gschneidner Jr K. A. (1999). J. Appl. Phys..

[cit29] França E. L. T., dos Santos A. O., Coelho A. A., da Silva L. M. (2016). J. Magn. Magn. Mater..

[cit30] Pecharsky V. K., Gschneidner K. A., Tsokol A. O. (2005). Rep. Prog. Phys..

[cit31] Gschneidner Jr K. A., Pecharsky V. K., Tsokol A. O. (2005). Rep. Prog. Phys..

[cit32] Phan M. H., Yu S. C. (2007). Magn. Magn. Mater..

[cit33] Dhahri M., Zaidi A., Cherif K., Dhahri J., Hlil E. K. (2017). J. Alloys Compd..

[cit34] Sun Y., Tong W., Zhang Y. (2001). J. Magn. Magn. Mater..

[cit35] Tegus O., Bruck E., Buschow K. H. J., de Boer F. R. (2002). Nature.

[cit36] Nam D. N. H., Dai N. V., Hong L. V., Phuc N. X., Yu S. C., Tachibana M., Takayama Muromachi E. (2008). J. Appl. Phys..

[cit37] Barik S. K., Krishnamoorthi C., Mahendiran R. (2011). J. Magn. Magn. Mater..

[cit38] Arrott A. (2008). J. Appl. Phys..

[cit39] Amaral J., Silva N., Amaral V. (2007). Appl. Phys. Lett..

[cit40] Tishin A. M. and Spichkin Y. I., The Magnetocaloric Effect and its Applications, IOP Publishing, 2003, Bristol

[cit41] Amaral J., Silva N., Amaral V. (2007). Appl. Phys. Lett..

[cit42] Cyrot M. , et al., Magnétisme I-Fondement, Presses universitaires de Grenoble, Grenoble, 1999

[cit43] Dhahri Ah., Jemmali M., Taibi K., Dhahri E., Hlil E. K. (2015). J. Alloys Compd..

